# Submolecular Resolution of β‑Sheet Plasticity: Decoding Mutations and PTMs in Protein Aggregation Disorders

**DOI:** 10.1021/acscentsci.5c00421

**Published:** 2025-05-16

**Authors:** Ruonan Wang, Zhongyi Jian, Yanlian Yang, Chen Wang, Lanlan Yu, Mingzhan Wang, Chenxuan Wang

**Affiliations:** † State Key Laboratory of Common Mechanism Research for Major Diseases, Department of Biophysics and Structural Biology, 12501 Institute of Basic Medical Sciences Chinese Academy of Medical Sciences, School of Basic Medicine Peking Union Medical College, Beijing 100005, P. R. China; ‡ CAS Key Laboratory of Biological Effects of Nanomaterials and Nanosafety, CAS Key Laboratory of Standardization and Measurement for Nanotechnology, CAS Center for Excellence in Nanoscience, National Center for Nanoscience and Technology, Beijing 100190, P. R. China; § Center of Super-Diamond and Advanced Films (COSDAF), Department of Materials Science and Engineering, 53025 City University of Hong Kong, Kowloon 999077, Hong Kong, P. R. China

## Abstract

The functional diversity of proteins often arises from the remodeling of conformational ensembles, particularly through mutations and post-translational modifications (PTMs). However, experimentally characterizing such ensembles remains challenging due to their heterogeneous and transient nature. Here, we report the determination of the conformational substates of β-sheets and the effect associated with mutations and PTMs in human islet amyloid polypeptide (hIAPP) via scanning tunneling microscopy (STM). Thanks to the ultrahigh resolution of STM, the β-sheets formed by the assembly of hIAPP were revealed to be conformationally diverse, including 17 types of conformational substates concomitant with 60 types of interconformation interactions. These conformational substates are highly heterogeneous in the folding structures but close in energy. Four mutations and PTMs were carried out with hIAPP to investigate the evolvability of the β-sheet assembly. Regulation effects accomplished by the mutations and PTMs on the conformational ensembles of β-sheets have been identified, including the number of conformational substates, the most probable substates, and the topography of the energetic landscapes of inter-β-strand interactions. Different types of variations show divergence in the influences on the β-sheet conformational ensembles, which is correlated with the divergent aggregation propensity. Our results highlight the plasticity of conformational ensembles upon mutations and PTMs.

## Graphical abstract

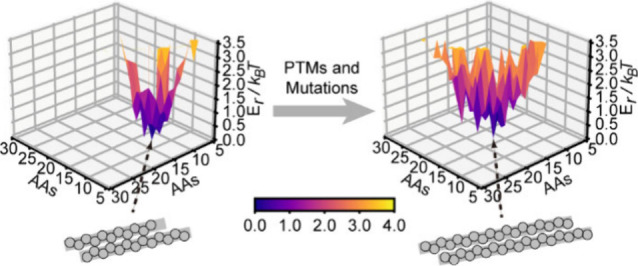

## Introduction

The noncovalent association of natural or artificial biological building blocks into supramolecular structures is essential for unveiling the mechanisms of biomolecular functions and for artificially constructing exquisite biomaterials.^1–3^ Among the diverse hierarchical architectures made by biomolecules, β-sheet fibrils formed from the self-assembly of β-strands have attracted great interest due to their relevance to the pathology of amyloidosis and its applications in biomaterial engineering.^4–6^ β-Sheet fibrils feature periodically parallel or antiparallel arrangement of β-strands where each β-strand is perpendicular to the direction of fibril elongation.^7^ Typically, β-strands in a single fibril are thought to be packed in a highly ordered manner and uniform in conformation.^8–10^ For example, the structure of the wild-type hIAPP-assembled fibril, an amyloidal species associated with the onset and progression of type 2 diabetes, shows that each monomer folds into an S-shaped conformation featuring three β-strands (residues 14–19, 26–31, and 35–36) (PDB 6ZRF) in cryo-EM characterizations.^11,12^ Interpeptide interactions stabilize the folding structure of hIAPP and lead to the periodic arrangement of hIAPP monomers into a protofilament.^11,12^

Mutations and PTMs play an important role in tailoring the interpeptide interactions and in altering the folding and assembly structure of β-sheet fibrils.^13–15^ For instance, a serine to glycine substitution at residue 20 (S20G) in hIAPP, a hereditary mutation linked to the early onset of type 2 diabetes, causes residues 15–37 of hIAPP S20G to adopt a hairpin-like conformation featuring four β-strands (residues 15–18, 21–23, 28–31, and 35–36) to form a protofilament (PDB 6ZRQ).^11,12^ The distinct structures between the wild-type and the mutant assemblies provide a straightforward lens for understanding the complexity of the pathological properties of β-sheet fibrils. However, recent high-resolution studies have indicated that such structural complexity of β-sheet fibrils is beyond the traditional view that proteins preserve a single, fixed structure with the lowest free energy and far from being well understood:^16–20^ (i) β-sheet fibrils exhibit a clear polymorphism in structure and β-strands from different β-sheet fibrils can fold into distinct conformations;^20–22^ (ii) even the β-strands from a single β-sheet fibril can exist as an ensemble of multiple coexisting conformations, instead of a fixed conformation.^23–25^ This structural plasticity has been increasingly recognized as a common feature in amyloid systems, with notable examples including Aβ fibrils in Alzheimer’s disease and α-synuclein assemblies in Parkinson’s disease, where conformational variations directly correlate with distinct pathological phenotypes and disease progression rates.^21,26^ The existence of a protein in multiple conformations increases the functional diversity of a protein, multiplying the diversity caused by the mutations and PTMs with sequence.^27–29^ Thus, important questions emerge: how do the heterogeneously coexisting conformations of β-sheets evolve? Particularly, how does the evolution of a conformational ensemble respond to the mutations and PTMs? The chaotropic nature of protein structures also motivates us to re-examine the effects associated with mutations and PTMs. Not only the lowest free energy structures but also the ensemble of metastable substates and the intermolecular interaction networks should be considered to evaluate the impacts of mutations and PTMs on structures.^21,24,25,30,31^

In this work, we aim to obtain a more comprehensive picture of mutations and PTMs in regulating the conformational ensembles of β-sheet fibrils. Four representative mutations and PTMs have been examined, including the disease-associated mutant (hIAPP S20G), the terminus-substituted mutant (an amide to carboxyl group substitution at the C-terminus in hIAPP, hIAPP COOH hereafter), the multiple-site mutant (an arginine to histidine substitution at residue 18 in rat islet amyloid polypeptide, rIAPP R18H hereafter), and the artificial mutant carrying PTM (phosphorylated serine at residue 20 in hIAPP, hIAPP S20p hereafter) (Figure 1A, Supporting Information section 1.1). We applied an STM imaging-based probability interpretation (SIPI) method,^24,25^ through which the simultaneously coexisting conformational substates in a single β-sheet fibril can be distinguished by the STM-based single-molecule imaging. Notably, compared to ensemble-averaging techniques like cryo-EM and NMR, the STM approach offers distinct advantages: high sensitivity enabling detection of low-population conformational substates by avoiding the average of heterogeneous structures.^24,25^ By comparing the conformational ensembles and intermolecular energy landscapes of the β-sheet assemblies formed by the hIAPP and its variants, the roles of mutations and PTMs in remodeling the conformational ensembles and intermolecular energy landscapes of β-sheets are portrayed, which provides a new perspective for studying the complicated relationship between the conformational ensembles and the function of protein assemblies.

**Figure 1:**
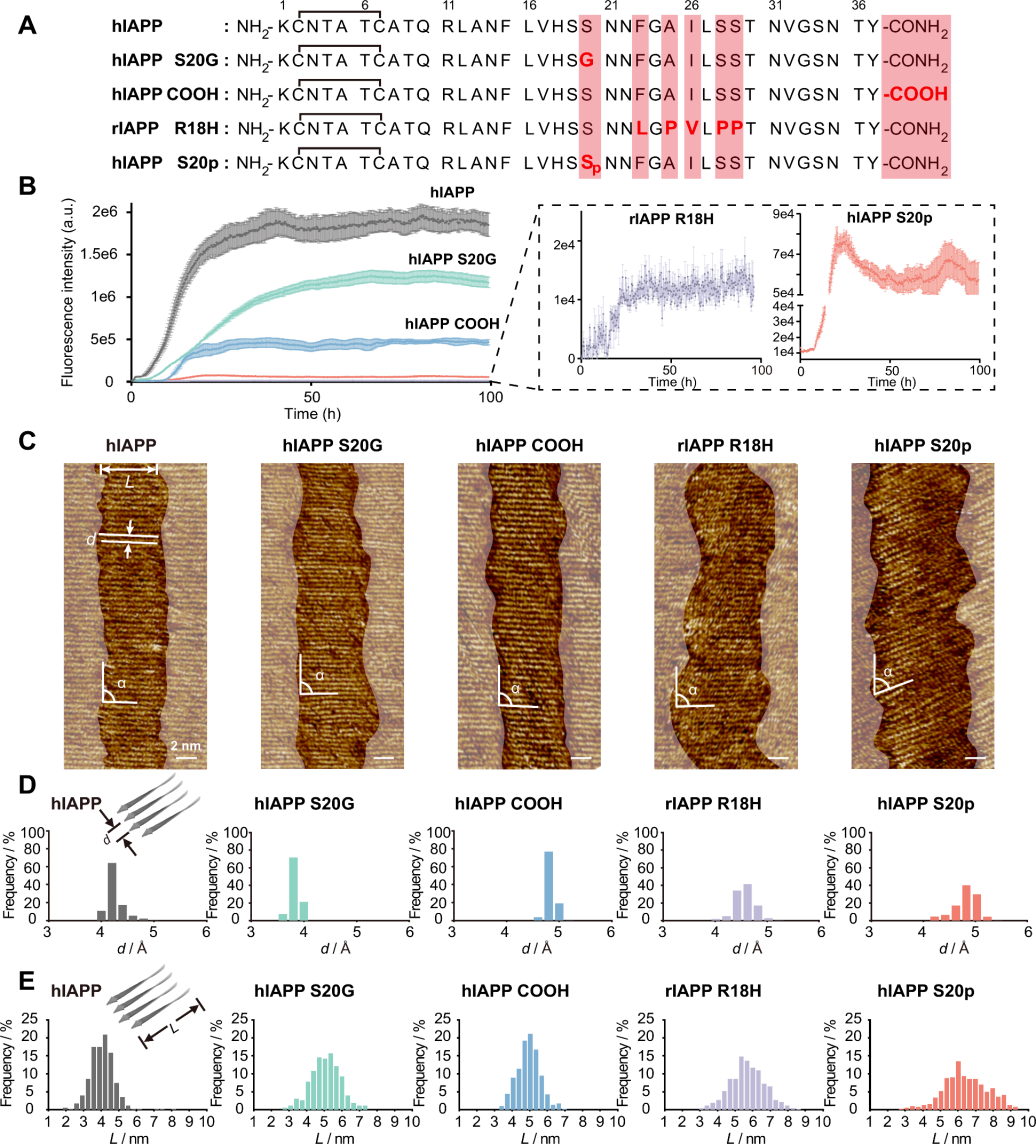
Assembly structures of the hIAPP and its variants. (A) The primary sequences of the hIAPP and its typical variants. (B) ThT fluorescence intensity is plotted over time. (C) Representative STM images of the β-sheet assembled by hIAPP and its variants. Tunneling conditions: tunneling current (*I*) = 198.4 pA, bias voltage (*V*_bias_) = 459.9 mV (hIAPP); *I* = 198.4 pA, *V*_bias_ = 499.9 mV (hIAPP S20G); *I* = 198.4 pA, *V*_bias_ = 500.0 mV (hIAPP COOH); *I* = 198.4 pA, *V*_bias_ = 499.9 mV (rIAPP R18H); and *I* = 198.4 pA, *V*_bias_ = 550.0 mV (hIAPP S20p). (D, E) Histograms of the separation between neighboring peptide strands (D) and the length of the peptide strands (E) from 500 peptides, respectively.

## Results and Discussion

### β-Sheet Structure Is the Common Feature of the Assemblies Formed by hIAPP and Its Variants

hIAPP is a 37-residue, C-terminally amidated peptide with a disulfide bond between cysteine residues 2 and 7.^12^ The β-sheet assembly of hIAPP into a fibril-like supramolecular structure was achieved by incubating 40 μM hIAPP in an aqueous solution at 37 °C over a time course of 96 h. The structural conversion of hIAPP has been verified by monitoring the thioflavin-T (ThT) fluorescence, an indicator for the formation of β-sheet-rich aggregates,^25^ where the ThT fluorescence reaches the plateau phase after 50 h (Figure 1B, Supporting Information section 1.2). The transmission electron microscopy (TEM) and Fourier-transform infrared (FTIR) spectroscopy analyses also confirm the structural conversion of hIAPP into a β-sheet assembly. TEM imaging indicates that the morphology of hIAPP-assembled fibrils reaches an equilibrium after a 72-h incubation, while FTIR spectra show characteristic bands of the β-sheet (the amide I band at 1625 cm^–1^) and β-turn (the amide I band at 1665 cm^–1^ and 1677 cm^–1^) (Figure S1).^32–34^ Likewise, the β-sheet fibrillization of the hIAPP variants, including hIAPP S20G, hIAPP COOH, rIAPP R18H, and hIAPP S20p, is also confirmed (Figures S2–S5). The β-sheet conformation of hIAPP and its variants was also verified by circular dichroism (CD) spectroscopy measurements (Figures S6–S10). The *K*-means clustering algorithm with the elbow method was carried out to analyze the intensity of ThT fluorescence at the plateau phase (Figure S11, Supporting Information section 1.3). Accordingly, hIAPP and its variants were categorized into two kinds: one showing strong-to-medium aggregation propensity, i.e., hIAPP, hIAPP S20G, and hIAPP COOH, while the other shows weak aggregation propensity, i.e., hIAPP S20p and rIAPP R18H.

To gain insights into the microscopic structures of the β-sheets formed by hIAPP and its variants, the samples were deposited on a freshly cleaved highly oriented pyrolytic graphite (HOPG) surface for STM-based single-molecule imaging. The β-sheet assemblies formed by hIAPP and its variants display lamella-like structures in the STM image where the bright stripes within a lamella correspond to the β-strand regions of peptides (Figure 1C, Figures S12–S17). The average separations between two neighboring β-strands were measured to be 4.2 ± 0.2 Å for hIAPP, 3.7 ± 0.1 Å for hIAPP S20G, 4.7 ± 0.1 Å for hIAPP COOH, 4.4 ± 0.2 Å for rIAPP R18H, and 4.7 ± 0.2 Å for hIAPP S20p, consistent with the characteristic interstrand spacing in the β-sheet peptide assembly (Figure 1D).^35,36^ As well-known, β-strands are arranged parallel or antiparallel to each other in a typical β-sheet structure.^7^ We then measured the angle between β-strand axes and the lamellae direction, α. Most of the α values are equal to ∼90°. However, hIAPP S20p is an exception, whose α value is 70°. The decreased α value observed with the hIAPP S20p assembly hints at the impacts of phosphorylation on tilting the interpeptide packing of the hIAPP β-strands. Besides, the β-strand length shows a clear susceptibility to the mutations and PTMs: it increases from 4.1 ± 0.7 nm for hIAPP, to 4.9 ± 0.8 nm for hIAPP S20G, to 4.8 ± 0.6 nm for hIAPP COOH, to 5.5 ± 1.0 nm for rIAPP R18H, and 6.4 ± 1.2 nm for hIAPP S20p (Figure 1E and Figures S18–S22).

### Mutations and PTMs of the hIAPP Differentiate the Coexisting Conformational Substates within the β-Sheets

Since the separation between two neighboring residues within one β-strand is 3.25 Å, the number of amino acid residues of the β-strand can be therefore deduced from the length of the β-strand.^37^ Based on the residue number, we categorized the total coexisting conformational substates of hIAPP and its variants into 24 distinct substates with the residue number of amino acids (varying from 6 to 29) in the β-strand. These substates are marked from *I* (the β-strand consisting of 6 residues) to *XXIV* (the β-strand consisting of 29 residues) (Figure 2A). The conformational ensemble of hIAPP β-strands includes 17 types of coexisting conformational substates as labeled from *I* to *XIV*, *XVII* to *XVIII*, and *XX*. The most probable substates are *VIII* (20.8%, 13 residues in the β-strand), *VII* (17.7%, 12 residues in the β-strand), and *VI* (17.3%, 11 residues in the β-strand) (Figure 2B and Figure S23A). There is no conformational substate that accounts for >25% of the total population of β-strands, while there exist minorly populated substates with a population smaller than 1%, such as the substates *I*, *II*, and *XX*. This feature indicates that the hIAPP in the assembled state conforms to intrinsically disordered proteins.^38^

**Figure 2:**
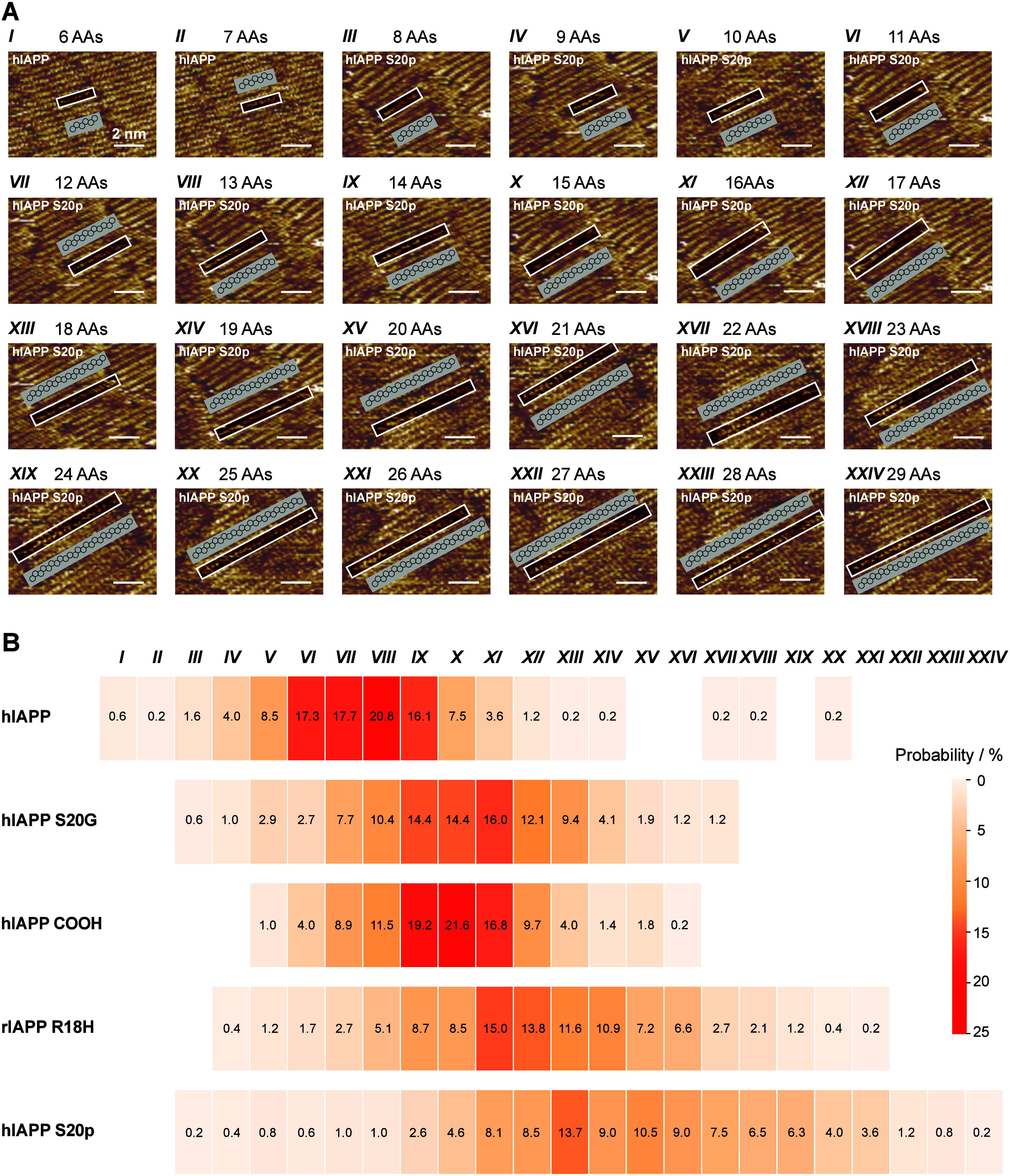
Mutations and PTMs differentiate the coexisting conformational substates within the β-sheets formed by hIAPP. (A) Representative STM images and structural models for each conformational substate in the assembly of the hIAPP and its variants. (B) Coexisting conformational substates identified from the β-sheets formed by hIAPP and its variants. The number indicates the proportion of the corresponding substate.

No predominant conformational substate exists in the assembly structure of the four variants of hIAPP either (Figure 2B), indicating their intrinsically disordered protein nature. However, it can be judged that mutations and PTMs in the hIAPP can exert distinct effects on differentiating the ensemble of the coexisting conformational substates. For hIAPP S20G and hIAPP COOH, the types of coexisting conformational substates decrease from 17 (hIAPP) to 15 and 12, respectively (Figure 2B, Figure S23B and S23C). Meanwhile, the number of β-strand residues identified from the most probable substates shifted from 11–13 in hIAPP to 14–16 in hIAPP S20G and hIAPP COOH (Figure 2B). By contrast, the types of coexisting conformational substates for hIAPP S20p increase to 22, while the most probable substate *XIII* corresponds to 18 residues (Figure 2B and Figure S23E). Last, as compared with the hIAPP, multiple-site mutant rIAPP R18H shows comparable types of coexisting conformational substates (18 types) but longer β-strand length in the most probable substates (16 residues in the substate *XI*) (Figure 2B and Figure S23D). Beyond the types of coexisting conformational substates and the number of β-strand residues, the proportion of each conformational substate is also changed with the mutations and PTMs (Figure 2B).

### Mutations and PTMs Regulate the Inter-β-strand Recognition of hIAPP

Each β-strand in a β-sheet assembly is tightly linked by inter-β-strand interactions, and thereby the conformational diversity of a β-sheet results in a high degree of complexity in the inter-β-strand interactions. For example, the conformational substate *VIII* of the hIAPP β-strands participates in the formation of 13 types of interactions among conformational substates, including the homogeneously interconformation interactions, e.g., *VIII–VIII* interactions, and heterogeneously interconformation interactions, e.g., *VIII–VI* interactions (Figure 3A). The frequencies of different types of interconformation interactions also vary from each other. To present the complex interactions among conformational substates, we coined a two-dimensional probability matrix showing the interactions among the conformational substates of hIAPP (Figure 3B); 60 types of interactions can be identified, while the highest proportions occur between the *VII* and *VIII* substates (8.6%) and between the *VIII* and *IX* substates (7.7%).

**Figure 3:**
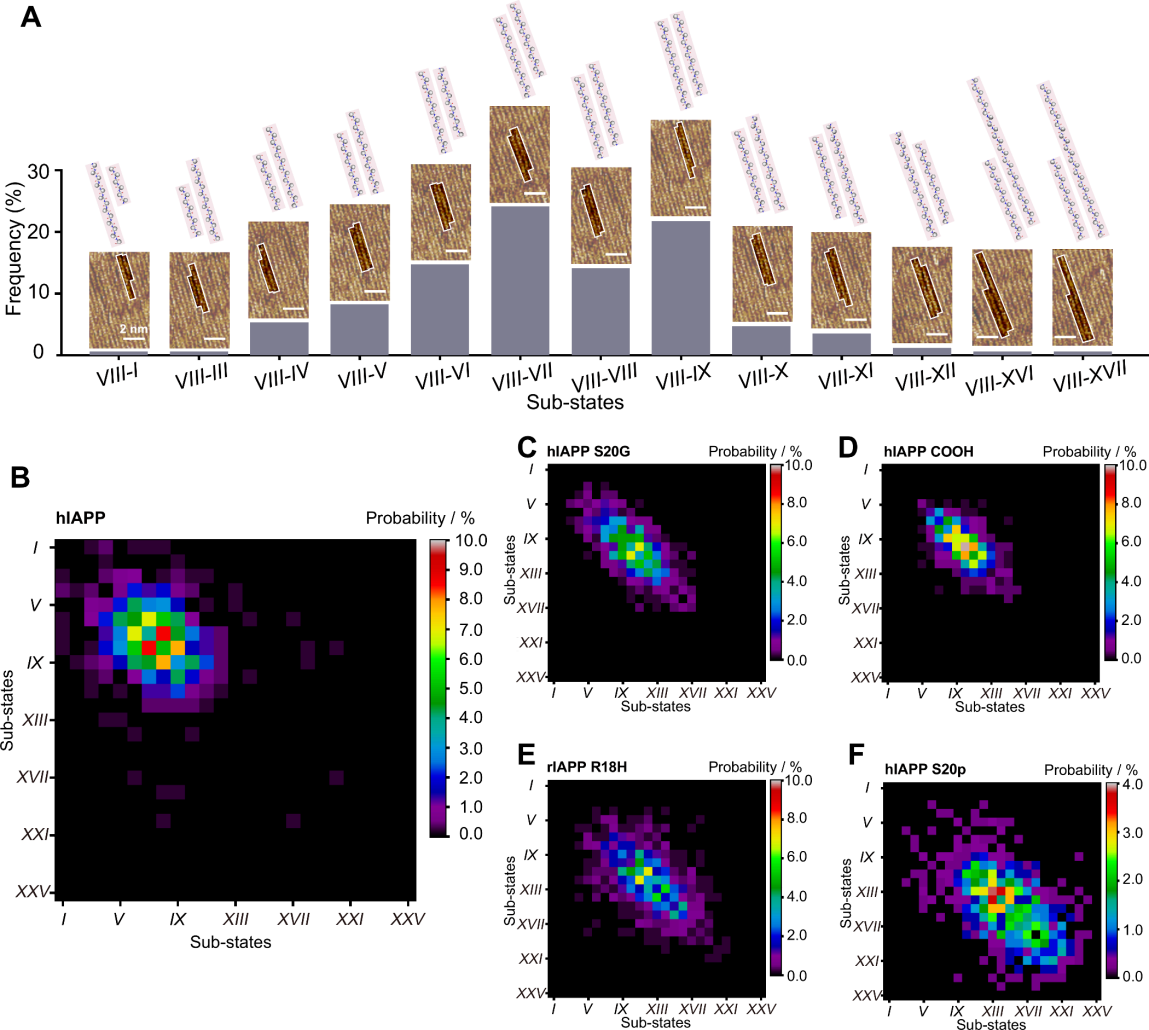
Mutations and PTMs change the inter-β-strand recognition of hIAPP. (A) Representative STM images show the interactions between the substate *VIII* and other conformational substates of the hIAPP. The histogram shows the frequency of the interactions between the substate *VIII* and other conformational substates, which was determined from 478 pairs of interpeptide interactions. Tunneling conditions: *V*_bias_ = 459.9 mV and *I* = 198.4 pA. (B-F) Two-dimensional probability matrices showing the probabilities of substate interactions in the peptide assemblies of hIAPP (B), hIAPP S20G (C), hIAPP COOH (D), rIAPP R18H (E) and hIAPP S20p (F).

To unveil the variation effects on the inter-β-strand recognition within a β-sheet, the probability matrices showing interactions among the conformational substates of the hIAPP variants are analyzed as well. Strikingly, the multiple-site mutation with hIAPP increases the type of interactions by 60% to 97 and the phosphorylation with hIAPP nearly doubles the type of interactions from 60 to 113 (Figures 3E and 3F), suggesting greatly enriched types of interactions and more complex intermolecular recognition. By comparison, hIAPP S20G and hIAPP COOH display 67 and 50 types of interactions, respectively, which are comparable to those of the wild-type hIAPP (Figures 3C and 3D). The divergent responses of inter-β-strand recognition upon mutations and PTMs are reminiscent of the two clusters in the ThT fluorescence intensity (Figure 1B). The comparison of these two sets of experiments implies a possible molecular scenario of peptide aggregation: more types of interconformation interactions existing in the β-sheet, e.g., rIAPP R18H and hIAPP S20p, indicate a lower specificity of inter-β-strand interactions and weaker peptide aggregation propensity, and vice versa.

Considering the central role of interchain hydrogen bond networks in maintaining the structural order of β-sheets, we propose that the differential impacts of mutations and PTMs on the conformational ensembles and the interactions between conformational substates reflect the influence of these chemical groups on the interchain hydrogen bonds of β-sheets.^39,40^ Note that these mutations and PTMs occur at different positions of the hIAPP, either in the β-strand-forming core region (corresponding to the 14–37 residues of hIAPP),^41^ i.e., hIAPP S20G, hIAPP S20p, and rIAPP R18H, or in the terminal region, i.e., hIAPP COOH. Thus, we discuss the impacts of these variations based on their positions. First, the β-sheet conformational ensemble is amenable to the alternation with the hydrogen bonding potential of the aggregation core region. Specifically, the hydration free energy of serine is −27.4 kJ/mol, roughly equal to that of glycine (−34.3 kJ/mol).^42,43^ The similarity in the magnitude of the hydration free energy indicates their comparable ability to form hydrogen bonds with main chains or solvents. By comparison, the phosphate group has unique characteristics, such as the large and negative hydration free energy (H_2_PO_4_^–^, −465 kJ/mol; PO_4_^3–^, −2765 kJ/mol),^44^ the large molecular volume, and the potential of forming electrostatic interactions, and therefore, the phosphorylation with the serine at residue 20 is presumed to disturb the hydrogen bonding network of the β-sheets to a much larger extent. Consequently, the serine to glycine substitution and the phosphorylation with serine result in divergent impacts on the conformational ensemble of the β-sheet. Additionally, the result of rIAPP R18H also highlights the importance of hydrogen bonding potential in governing the conformational ensemble of the β-sheet. The major difference between rIAPP R18H and hIAPP is the three prolines at residues 25, 28, and 29 of rIAPP R18H. Proline is devoid of the NH group required for interchain hydrogen bonding and is known as a β-sheet breaker.^45^ Correspondingly, the β-sheet of rIAPP R18H exhibits more types of interconformation interactions compared to that of hIAPP. Second, the susceptibility to the hydrogen bonding potential appears weak in the terminal region. Although the hydration free energy of carboxylate (CH_3_COO^–^, −395 kJ/mol) is more favorable than that of amide (CH_3_CONH_2_, −39.9 kJ/mol),^44,46,47^ the amide to carboxyl substitution at the C-terminus of hIAPP affects little the conformational ensemble of the β-sheet. This may result from its deviation from the β-strand-forming core region of hIAPP.

### Mutations and PTMs Remodel the Energetic Landscapes of Inter-β-strand Recognition in a β-Sheet

To understand the inter-β-strand interactions from the perspective of energy, the relative free energy differences between distinct interactions between conformational substates identified from the β-sheets were analyzed via the SIPI method.^24,25^ For the β-sheet system at equilibrium, the type of interconformation interaction with the highest probability is defined as the lowest energy state (*E*_0_). The relative energy difference (Δ*E*_*i*_) between the interaction type *i* (*E*_i_) and the interaction type with the lowest energy state can be calculated according to the ratio of their probabilities *P*_i_ and *P*_0_ via the Maxwell–Boltzmann distribution:^48^

1 $${\Delta E_{i}=E}_{i}-E_{0}=-k_{B}T\ln\left(\frac{P_{i}}{P_{0}}\right)$$

where *k*_B_ is the Boltzmann constant, and *T* is the absolute temperature of the system. For the β-sheets formed by hIAPP, the lowest-energy interaction *VII–VIII* is taken as the reference state and the Δ*E*_*i*_ between the interaction *VII–VIII* and other specific interactions is deduced, so a full energetic landscape showing the interaction between conformational substates can be constructed. As shown in Figure 4A, the energetic landscape of the interconformation interactions in a hIAPP β-sheet displays a funnel shape. The Δ*E*_*i*_ maximum is 3.7 *k*_B_ *T*, corresponding to the depth of the energetic funnel. Different types of interactions between conformational substates are arranged in a hierarchy: the energy basin (the *VII–VIII* interaction and the *VIII–VIII* interaction), the intermediate energy level (the *III*–*VI* interaction and the *VIII*–*XI* interaction), and the high-energy level (the *I*–*VIII* interaction and the *III*–*VIII* interaction).

**Figure 4:**
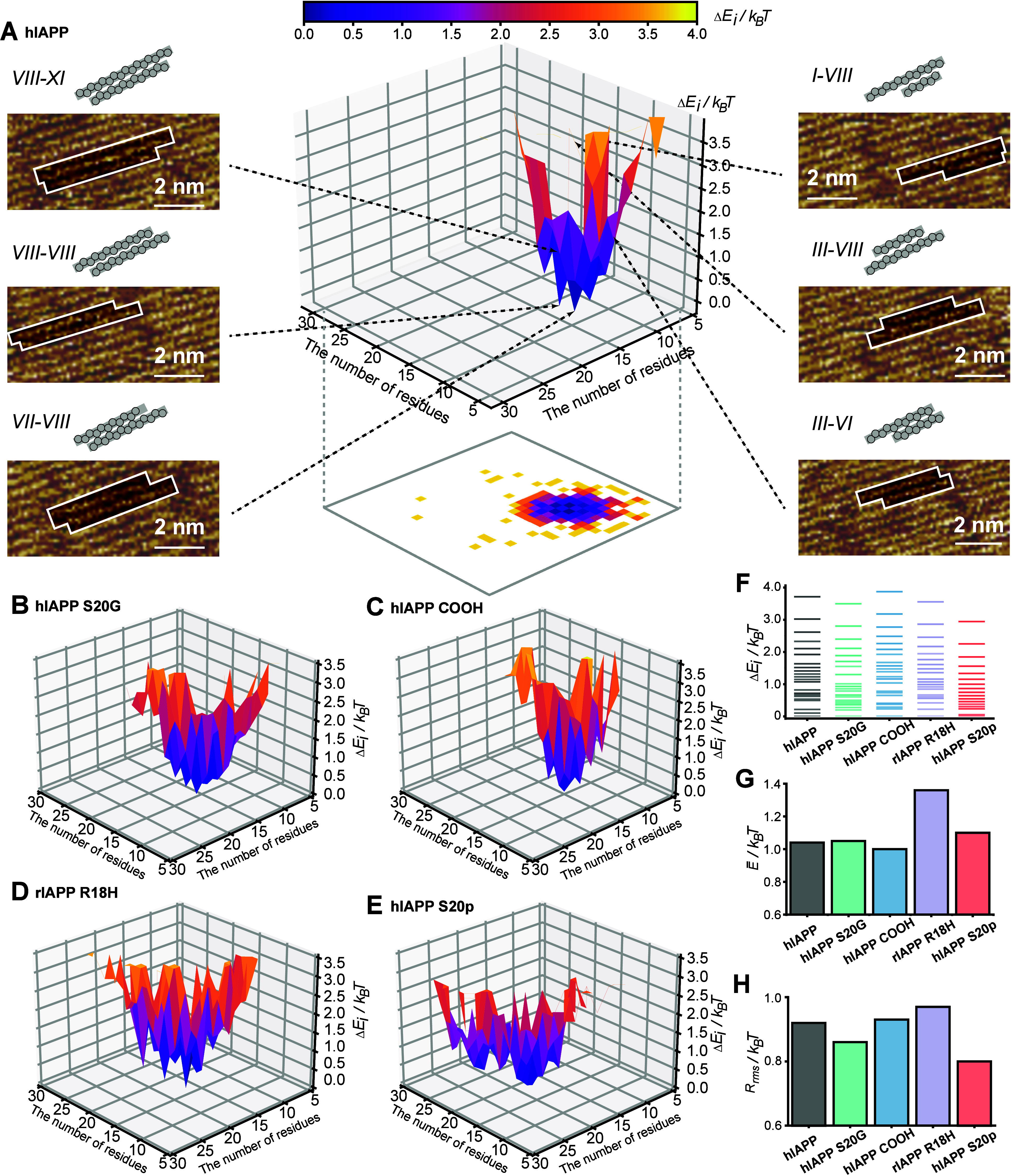
Mutations and PTMs remodel the energetic landscapes of inter-β-strand recognition in a β-sheet. (A) The three-dimensional energy landscape of the interconformation interactions for the β-sheet formed by the hIAPP and representative STM images showing the inter-β-strand interactions. Tunneling conditions: *V*_bias_ = 459.9 mV and *I* = 198.4 pA. (B-E) The three-dimensional energy landscapes of the interconformation interactions for the β-sheets formed by hIAPP COOH (B), hIAPP S20G (C), rIAPP R18H (D), and hIAPP S20p (E), respectively. (F) The distribution of the relative free energy difference for the β-sheets formed by hIAPP and its variants. The lowest *E*_i_ is set to be 0. (G, H) The weighted average energy (G) and the root-mean-square roughness (H) calculated for the β-sheets formed by hIAPP and its variants.

To investigate the impacts of mutations and PTMs on modulating the energetic landscapes of the intermolecular recognition, the inter-β-strand interactions within the β-sheets formed by variants were analyzed. Variations exhibit a profound influence on reformatting the topography of energetic landscapes (Figures 4B-4E). The range of the Δ*E*_*i*_ distribution is smaller than 4.0 *k*_B_ *T* (Figure 4F). Taking the lowest-energy interaction as the reference state, the weighted average energy (*E̅*) and the root-mean-square roughness (*R*_*rms*_) of the system were deduced as^49^

2 $$\mathrm{E̅}=\int_{0}^{\infty }{\Delta E}_{i}P_{i}d{\Delta E}_{i}$$

3 $$R_{rms}=\sqrt{\int_{0}^{\infty }{\left({\Delta E}_{i}-\mathrm{E̅}\right)}^{2}P_{i}d{\Delta E}_{i}}$$

For the energetic landscapes of hIAPP and variants, the fluctuations in the magnitude of *E̅* and *R*_*rms*_ are limited to a narrow range, ∼1.0 *k*_B_ *T*, close to a typical van der Waals bond (Figures 4G and 4H).^50^ The comparison of the energetic landscapes between hIAPP and its variants leads to two intriguing observations. First, the *E̅* values of rIAPP R18H β-sheets (1.4 *k*_B_ *T*) and hIAPP S20p (1.1 *k*_B_ *T*) are higher than those of other mutants, indicating that more high-energy interactions are involved in the β-sheet formation of these two variants (Figure 4G). Second, although hIAPP S20p and rIAPP R18H show similarity in several aspects, including the weak aggregation propensity, the large numbers of coexisting conformational substates, and the increased types of interconformation interactions, they might adopt distinguishing mechanisms to form the heterogeneous interconformation interactions. For hIAPP S20p, both the *R*_*rms*_ and the Δ*E*_*i*_ maximum are smaller than those of other peptides (Figures 4F and 4H). The smooth topography of the energy funnel adopted by the hIAPP S20p β-sheet facilitates the interconversion between different types of interconformation interactions due to the absence of a high energy barrier. By contrast, the *R*_*rms*_ of rIAPP R18H is higher than those of other variants, indicating that the energy funnel of the rIAPP R18H β-sheet is riddled with more energetic traps to stabilize the metastable substates relative to the β-sheets formed by others (Figure 4H). Our analysis reveals that mutations and PTMs can profoundly remodel the energetic landscapes of interconformation interactions within the β-sheets formed by hIAPP.

Our observations imply that the β-sheet assembly serves as an essential mechanism by which nature increases the conformational diversity of proteins, facilitating the divergence of alternative structures and functions. Each mutation and PTM displays more than 10 types of distinct conformational substates, and each conformational substate participates in the formation of ∼10 types of heterogeneous inter-β-strand interactions to construct a β-sheet. Therefore, the resulting structural diversity from the β-sheet assembly increases by approximately 2 orders of magnitude compared to the sequence diversity, making up large repertoires on which selection acts. Two characteristics are highlighted by the repertoires of β-sheet substates. First, the structures of coexisting conformational substates in a β-sheet formed by hIAPP or its variants are highly heterogeneous, where the residue number of amino acid varies from 6 to 29 in a β-strand. Second, these conformationally heterogeneous substates are close in energy, as manifested by their comparable *E̅* and *R*_*rms*_ magnitude in the intermolecular energetic landscapes. Collectively, in contrast with the conventional opinion that the β-sheet assembly is highly ordered in the supramolecular structures, our results indeed reveal the disorder, conformational diversity, and evolvability of the β-sheet assemblies.

## Conclusion

In brief, we report the use of the SIPI method targeting a thorough understanding of the conformational ensemble of β-sheets of the hIAPP-based assembly. We particularly highlight the effect of mutations and PTMs in the primary structure of hIAPP on the conformational ensemble of the β-sheets formed from the hIAPP-based assembly. The β-sheets formed through the assembly of hIAPP are conformationally diverse, including 17 types of coexisting conformational substates and 60 types of interconformation interactions. The four types of mutations and PTMs examined in this study were observed to implement profound regulations on the ensemble of coexisting conformational substates, changing the number of coexisting conformational substates and the most probable substates. These alternations result in differences in the inter-β-strand recognition and the energetic landscape of interconformation interactions. The distinguishing responses of β-sheet formed by hIAPP toward different mutations and PTMs reflect the distinct potential of chemical groups in tailoring interchain interaction networks, providing single-molecular insight into understanding their divergent aggregation propensity. This effort would illuminate a new direction for rethinking the role of mutations and PTMs in the pathological mechanisms of disease, as well as the rational design of protein/peptide-based supramolecular architectures and molecular machines.

## Materials and Methods

### Reconstitution of Peptide Assemblies in Solution

The peptides were synthesized and purified by Anhui Guoping Pharmaceutical Co., Ltd., with a purity above 98%, as verified by high-performance liquid chromatography and mass spectroscopy. To unfold the peptides, 1,1,1,3,3,3-hexafluoro-2-propanol (HFIP, Innochem) was utilized and subsequently evaporated by using nitrogen gas. The peptides were then refolded and assembled by dissolving them in 100 μM TEA buffer at pH 6 at a concentration of 40 μM. The peptide assemblies were equilibrated in solution at 37 °C with shaking at 210 rpm for 72 h.

### Peptide Concentration Determination

A 6 M guanidinium chloride aqueous solution was used to disrupt the folding structures of the peptides. The concentrations of the peptides were determined by using a UV–vis spectrophotometer (PerkinElmer, USA). The UV absorbance (*A*) of the peptides at the wavelength of 280 nm was correlated with the molar extinction coefficient of the peptides (*ε*), the optical path length of the cell (*L*), and the peptide concentration (*C*) according to the Beer–Lambert law equation:^51^

4 A=εLC

### FTIR Spectroscopy Measurements

A 20 μL aliquot of the peptide solution was deposited onto a calcium fluoride window. Once the peptide solutions were dried, the FTIR spectra were recorded by using a PerkinElmer Spectrum One FTIR Spectrometer (Waltham, Massachusetts, USA). The spectral range covered wavenumbers from 400 to 4500 cm^–1^ with an increment of 2 cm^–1^. PeakFit software (Version 4.12, San Jose, CA, USA) and Origin software (Version 9.1, Northampton, MA, USA) were utilized to deconvolute and analyze the second-order derivatives of the FTIR spectra.

### TEM Measurements

To observe the peptide assemblies, 10 μL of the peptide solution was deposited onto a 200 mesh Formvar carbon-coated copper TEM grid and allowed to settle for 2 min at room temperature. The excess solution was removed by using filter paper, and the samples were dried for 2 h at room temperature. Staining was performed by using 1% phosphotungstic acid for 30 s. After being rinsed with Milli-Q water three times, the grids were thoroughly dried before TEM characterization. A Hitachi H-7650 transmission electron microscope (Hitachi, Tokyo, Japan) was used to visualize the topological features of the peptide assemblies.

### CD Analysis

A Jasco J-1500 circular dichroism spectropolarimeter system (Japan) was used to collect the CD spectra of hIAPP and its variants at room temperature. The path length of the cuvette used was 1 mm. The spectra were collected within a wavelength range of 185 to 260 nm at a scan speed of 50 nm/min. The bandwidth was set as 2 nm, and the digital integration time was 1 s. The CD signals collected from the samples were corrected by subtracting the CD signals of the solvent alone.

### ThT Fluorescence

To monitor the aggregation kinetics of β-sheet fibril formation, a ThT fluorescence assay was performed. ThT stock solution was prepared in deionized water and filtered through a 0.22 μm membrane to remove particulate matter. The final assay mixtures contained 50 μM ThT and 40 μM peptide in 100 μM TEA buffer at pH 6, with a total reaction volume of 200 μL. The mixtures were loaded into a black-bottom, clear-top 96-well plate, and each well was sealed with a clear adhesive film to prevent evaporation during incubation. Fluorescence measurements were performed on the Synergy H1 microplate reader, using excitation and emission wavelengths of 450 and 485 nm, respectively. The plate was incubated at 37 °C with moderate shaking, and fluorescence intensity was recorded every 15 min over a period of 100 h. Control experiments were conducted using the same conditions but without the peptide to account for the background ThT fluorescence.

### *K*-Means Clustering

*K*-means produces exactly *k* different clusters of the greatest possible distinction and partitions the peptides into *k* clusters in which each peptide belongs to the cluster with the nearest mean. The objective of cluster *k* was computed from the fluorescence intensity by minimizing total intracluster variance (the squared error function):^52^

5 $$J=\overset{k}{\underset{j=1}{\sum }}\overset{n}{\underset{i=1}{\sum }}{∥{x_{i}}^{\left(j\right)}-c_{j}∥}^{2}$$

where *J* is the objection function, *k* is the number of clusters, and *n* is the number of cases. The distance function is ∥*x*_*i*_^(*j*)^ – *c*_*j*_∥^2^, where *x*_*i*_ is case *i,* and *c*_*j*_ is the centroid for cluster *j*. For clarity, the algorithm procedure of *K*-means clustering with the elbow method can be described simply as follows: (1) Cluster the data into *k* groups where *k* is predefined. (2) Select *k* points at random as cluster centers. (3) Assign objects to their closest cluster center according to the *Euclidean* distance function. (4) Calculate the centroid or mean of all objects in each cluster. (5) Repeat steps 2, 3, and 4 until the same points are assigned to each cluster in consecutive rounds. (6) Plot the log total within the sum of squares against *k* for a sequence of values *k* = 1, 2, ..., *k*_*max*_. (7) Choose *k* at the elbow of the curve, where the line exhibits a change of slope.

### Single-Molecule Imaging by STM

A 10 μL aliquot of the peptide solution was deposited onto the freshly cleaved surface of HOPG, which was followed by natural drying. Under ambient conditions, single-molecule imaging experiments were conducted using a Nanoscope IIIa scanning probe microscope (SPM) system in constant-current mode (Bruker, USA). STM tips were fabricated from a Pt/Ir (80/20) wire. The STM experiments were independently repeated using different samples and tips.

### Interpeptide Interaction Analysis

The lengths of the peptide strands (*N* > 500) in the STM images were measured by using Nanoscope software (Bruker, USA). Assignments of the conformational substates were determined from the β-strand lengths. The neighboring peptide conformations for each conformational substate were identified from the STM images and counted for the statistical analysis of interpeptide interactions. The determination and statistical analysis can either be completed by manual measurement or automated using image software.

## Supplementary Material



## Notes

Designing the artificial mutant hIAPP S20p; Aggregation kinetics of hIAPP S20G; Performance of *K*-means clustering; FTIR spectroscopy, TEM images, and CD spectroscopy of peptide assemblies; *K*-means clustering algorithm with the elbow method; Details of STM experiments and analyses, including STM images of assemblies formed by hIAPP and its variants on HOPG surfaces; Length distribution of the peptide β strands; Representative STM images for each conformational substate. (PDF)
