# Supplementary material for: Submolecular Resolution of β‑Sheet Plasticity: Decoding Mutations and PTMs in Protein Aggregation Disorders
Source: ACS Cent Sci. 2025 May 16;11(6):927–37. doi: 10.1021/acscentsci.5c00421 (PMC12203261; doi:10.1021/acscentsci.5c00421)
Supplement: Supplementary file 1 [file oc5c00421_si_001.pdf]

Supporting Information

Sub-Molecular Resolution of β-Sheet Plasticity: Decoding Mutations and PTMs in Protein Aggregation Disorders

Ruonan Wang[1,#], Zhongyi Jian[1,#], Yanlian Yang[2], Chen Wang[2], Lanlan Yu[1,], Mingzhan Wang[3,*], and Chenxuan Wang[1,*]*

1 State Key Laboratory of Common Mechanism Research for Major Diseases, Department of Biophysics and Structural Biology, Institute of Basic Medical Sciences Chinese Academy of Medical Sciences, School of Basic Medicine Peking Union Medical College, Beijing 100005, P. R. China

2 CAS Key Laboratory of Biological Effects of Nanomaterials and Nanosafety, CAS Key Laboratory of Standardization and Measurement for Nanotechnology, CAS Center for Excellence in Nanoscience, National Center for Nanoscience and Technology, Beijing 100190, P. R. China

3 Center of Super-Diamond and Advanced Films (COSDAF), Department of Materials Science and Engineering, City University of Hong Kong, Kowloon 999077, Hong Kong, P. R. China

[‡] R.W. and Z.J. contributed equally to this work.

[*] Corresponding authors.

Prof. Chenxuan Wang, email: wangcx@ibms.pumc.edu.cn;

Prof. Mingzhan Wang, email: mwang552@cityu.edu.hk;

Prof. Lanlan Yu, email: yull@ibms.pumc.edu.cn.

1.1 The rationale for designing the artificial mutant hIAPP S20p

Mass spectrometry (MS)-based identification and quantification remains one of the most powerful approaches for studying PTMs. Among these, MS/MS fragmentation spectra provide direct and robust evidence for localizing PTM sites. However, hIAPP is a small peptide of approximately 3 kDa, which presents substantial analytical challenges (*J Bacteriol*. 2022; 204 (1): e0035321; *J Am Soc Mass Spectrom*. 2015; 26 (12): 1981-1991). Due to its low ionization efficiency, weak signal intensity, and limited fragmentation patterns, it is difficult to achieve confident site localization for PTMs. Currently, the only well-established PTM of hIAPP reported in protein databases is C-terminal amidation, and there is a lack of systematic studies specifically addressing phosphorylation at Ser20. Therefore, the precise pathological role of Ser20 phosphorylation in the context of type 2 diabetes remains unclear.

Nonetheless, Ser20 is located in the aggregation-prone core of hIAPP. The addition of a bulky, negatively charged phosphate group at this position is likely to disrupt local β-sheet structures or alter aggregation kinetics, potentially modulating the fibrillization process. Thus, phosphorylation at Ser20 may influence hIAPP aggregation behavior and could play a regulatory role in its pathological effects in type 2 diabetes. This hypothesis is supported by our experimental observations (**Figure 1B**), suggesting that this modification may have potential biological significance.

Furthermore, this artificial modification was engineered to probe a central question: how do point mutations and PTMs at the same residue influence the conformational ensembles of β-sheets? To address this, we selected the serine-to-glycine substitution and serine phosphorylation at residue 20. Compared to S20G, the phosphate group exhibits unique characteristics, including its large and negative hydration free energy ($H_2PO_4^-$, -465 kJ/mol; PO_4^{3-}, -2765 kJ/mol), significant molecular volume, and capacity for electrostatic interactions. Consequently, phosphorylation at Ser20 is predicted to disrupt the hydrogen bonding network of β-sheets to a far greater extent. Although S20 phosphorylation is synthetic, our data uncover universal biophysical principles governing PTM-amyloid relationships, providing a foundation for understanding how post-translational modifications regulate β-sheet conformational ensembles.

1.2 Aggregation kinetics of hIAPP S20G

Our ThT fluorescence assays reveal slower aggregation kinetics of the S20G mutant compared to earlier reports, which we attribute to methodological variations in sample preparation. Specifically, our experimental protocol utilized a distinct buffer system (100 μM TEA, pH 6.0) and incubation conditions (40 μM peptide, 50 μM ThT, 37 °C), whereas previous studies employed 10 μM peptide in 25 mM sodium phosphate buffer (pH 6.8) supplemented with 2% (v/v) DMSO at 30 °C (Nature Communications 2022, 13:1040). In addition, various factors such as pH, metal ion levels and interactions with other proteins may also modulate hIAPP aggregation behavior (Communications Biology 2024, 7, 776). Additionally, our selection of a low-ionic-strength buffer was necessitated by the requirements of STM experiments, as high-salt buffers can interfere with atomic-scale imaging resolution.

1.3 Performance of *K*-means clustering

We employed the K-means clustering algorithm to analyze ThT fluorescence intensity data for evaluating the aggregation propensity of hIAPP and its variants. The optimal cluster number (k = 2) was determined using the elbow method, as detailed in **MATERIALS AND METHODS**. The key statistical criterion was derived from the inflection point in the total within-cluster sum of squares (WSS) plot (**Figure S11**). As shown by the sharp WSS decrease from k = 1 to 2 followed by a plateau (k = 3, 4), the optimal cluster number was unambiguously identified at k = 2. This bipartite classification separates variants into two groups: 1) strong-to-moderate aggregation propensity (fluorescence intensity: hIAPP, hIAPP S20G and hIAPP COOH) and 2) weak aggregation propensity (rIAPP R18H and hIAPP S20p), as shown in **Figure S11**. To validate clustering robustness, we performed four experimental replicates. The high data reproducibility across different wells (**Figure 1B**) confirms the reliability of this classification framework.

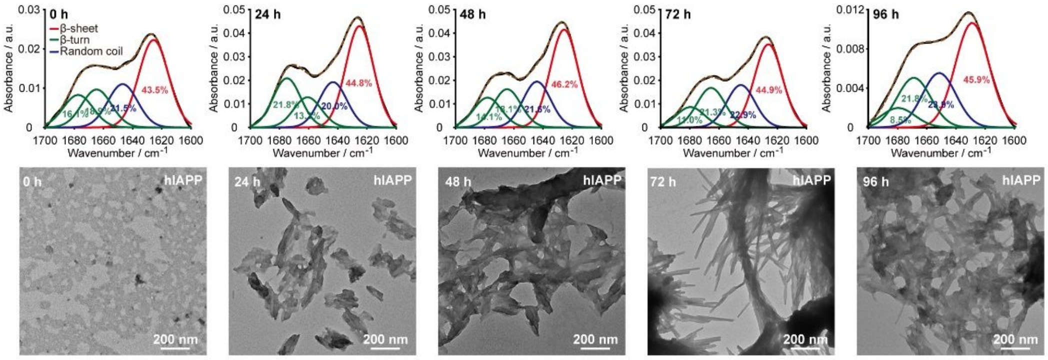

Figure S1. hIAPP β-sheet assembly. The second derivative FTIR spectra and TEM images of hIAPP peptide at different equilibration times (0, 24 h, 48 h, 72 h, 96 h, respectively). FTIR: black solid line, experimental curve; brown dashed line, fitting curve; red peaks, β-sheet component; green peaks, β-turn component; blue peaks, random coil component.

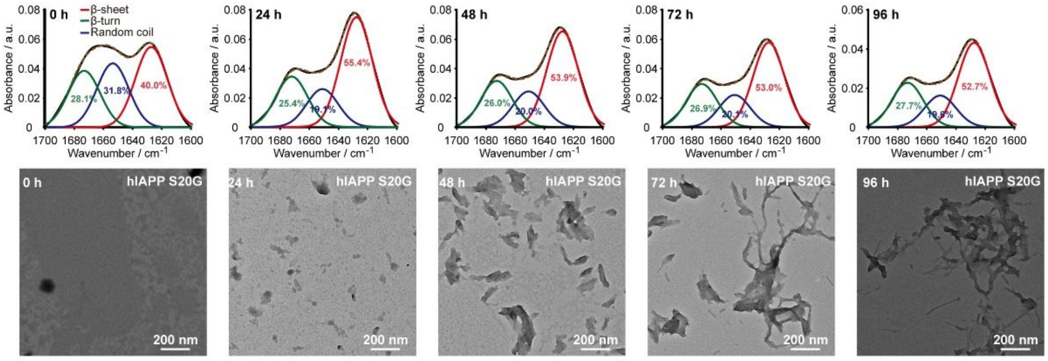

Figure S2. hIAPP S20G β-sheet assembly. The second derivative FTIR spectra and TEM images of hIAPP S20G peptide at different equilibration times (0, 24 h, 48 h, 72 h, 96 h, respectively). FTIR: black solid line, experimental curve; brown dashed line, fitting curve; red peaks, β-sheet component; green peaks, β-turn component; blue peaks, random coil component.

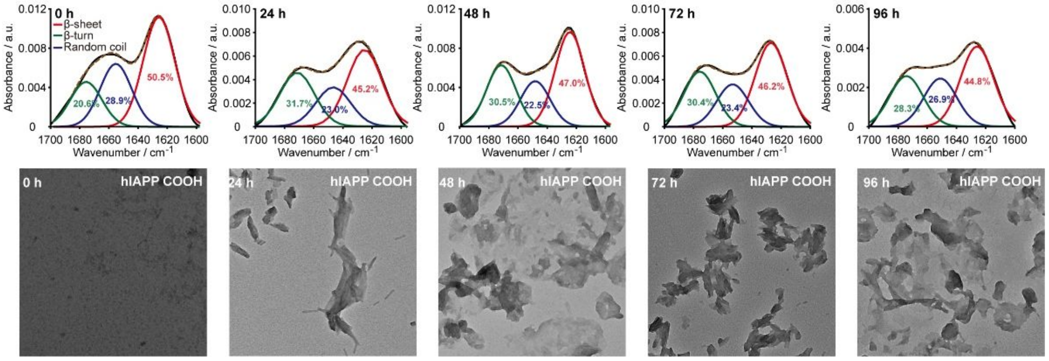

Figure S3. hIAPP COOH β-sheet assembly. The second derivative FTIR spectra and TEM images of hIAPP COOH peptide at different equilibration times (0, 24 h, 48 h, 72 h, 96 h, respectively). FTIR: black solid line, experimental curve; brown dashed line, fitting curve; red peaks, β-sheet component; green peaks, β-turn component; blue peaks, random coil component.

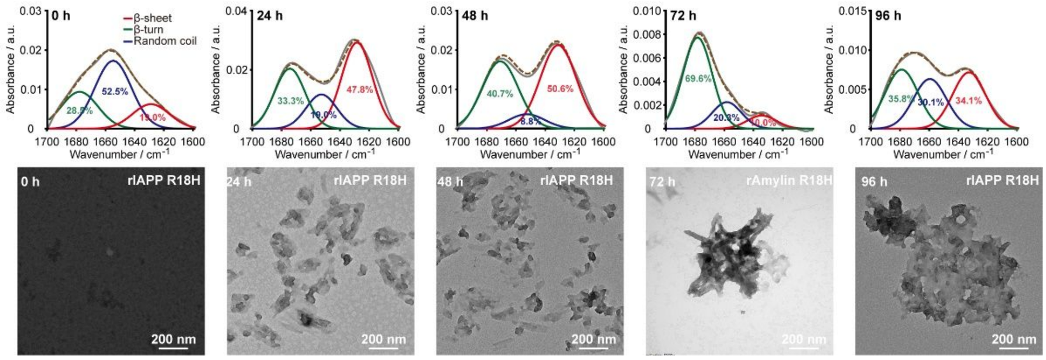

Figure S4. rIAPP R18H β-sheet assembly. The second derivative FTIR spectra and TEM images of rIAPP R18H peptide at different equilibration times (0, 24 h, 48 h, 72 h, 96 h, respectively). FTIR: black solid line, experimental curve; brown dashed line, fitting curve; red peaks, β-sheet component; green peaks, β-turn component; blue peaks, random coil component.

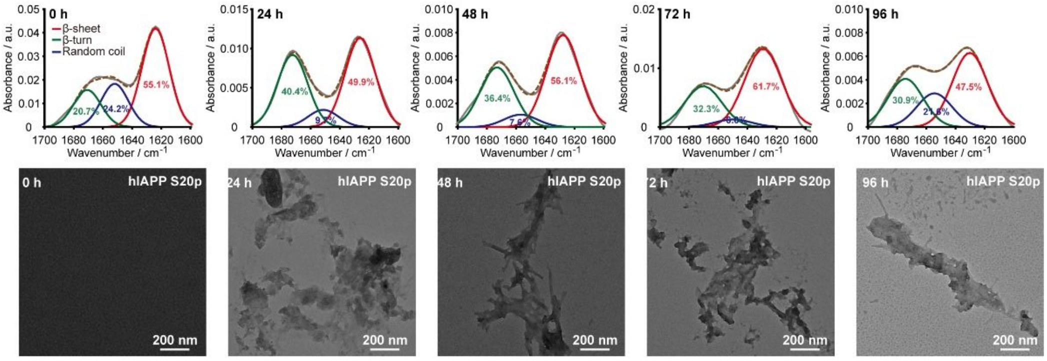

Figure S5. hIAPP S20p β-sheet assembly. The second derivative FTIR spectra and TEM images of hIAPP S20p peptide at different equilibration times (0, 24 h, 48 h, 72 h, 96 h, respectively). FTIR: black solid line, experimental curve; brown dashed line, fitting curve; red peaks, β-sheet component; green peaks, β-turn component; blue peaks, random coil component.

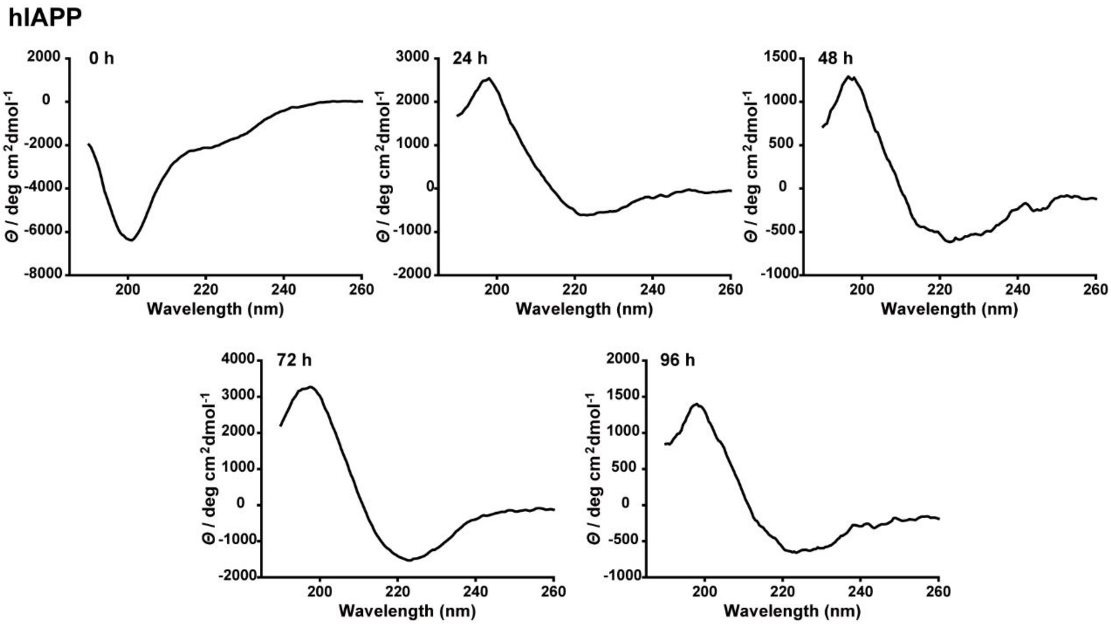

Figure S6. CD spectra of hIAPP at various equilibration times.

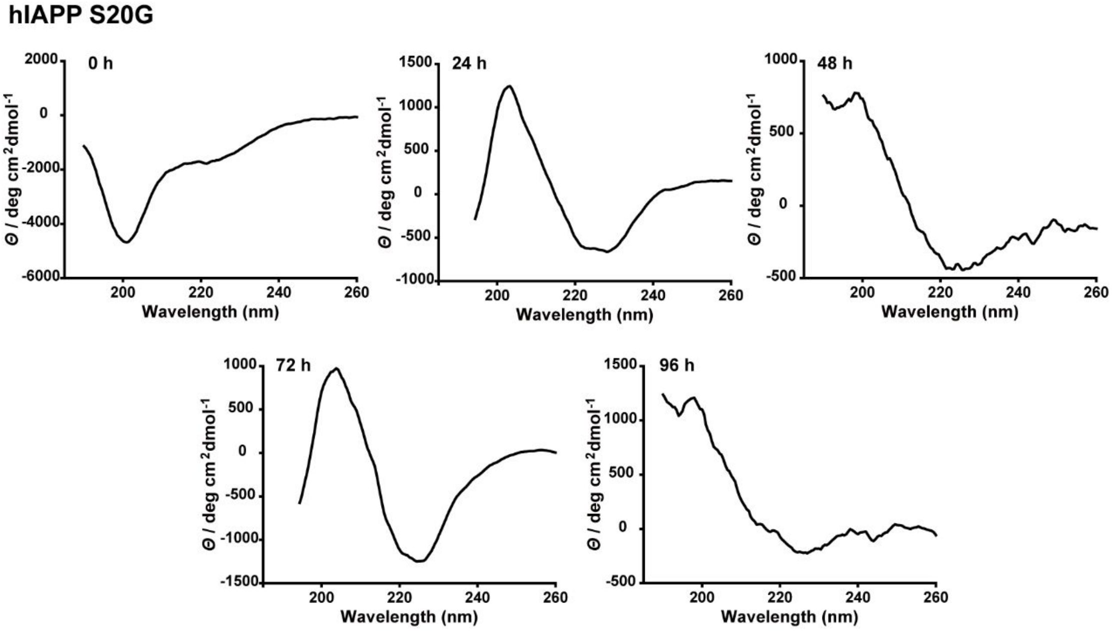

Figure S7. CD spectra of hIAPP S20G at various equilibration times.

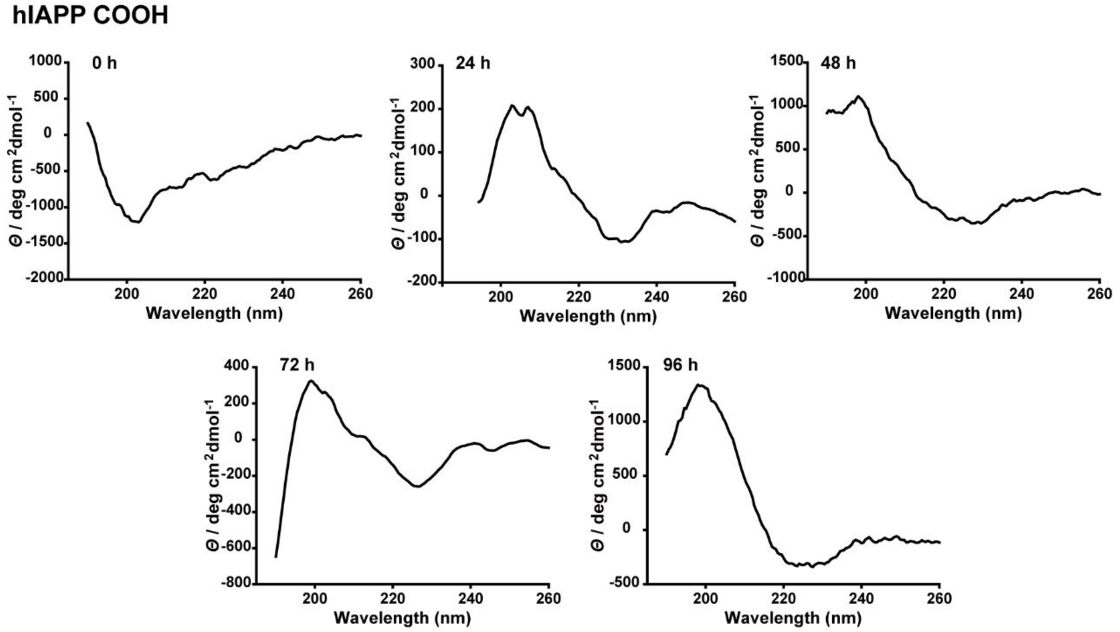

Figure S8. CD spectra of hIAPP COOH at various equilibration times.

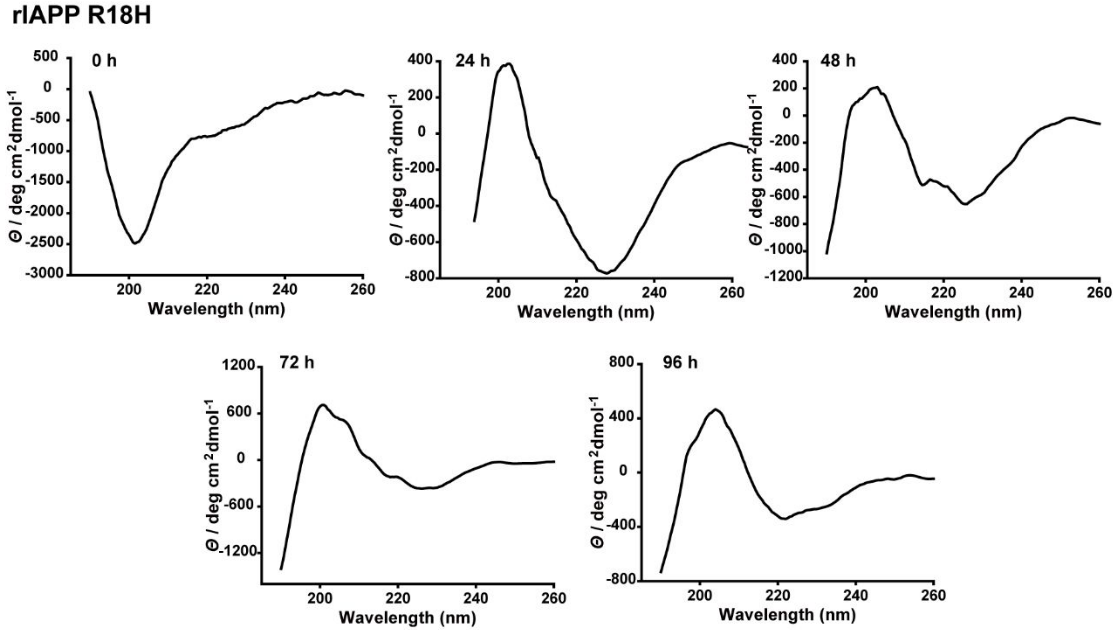

Figure S9. CD spectra of rIAPP R18H at various equilibration times.

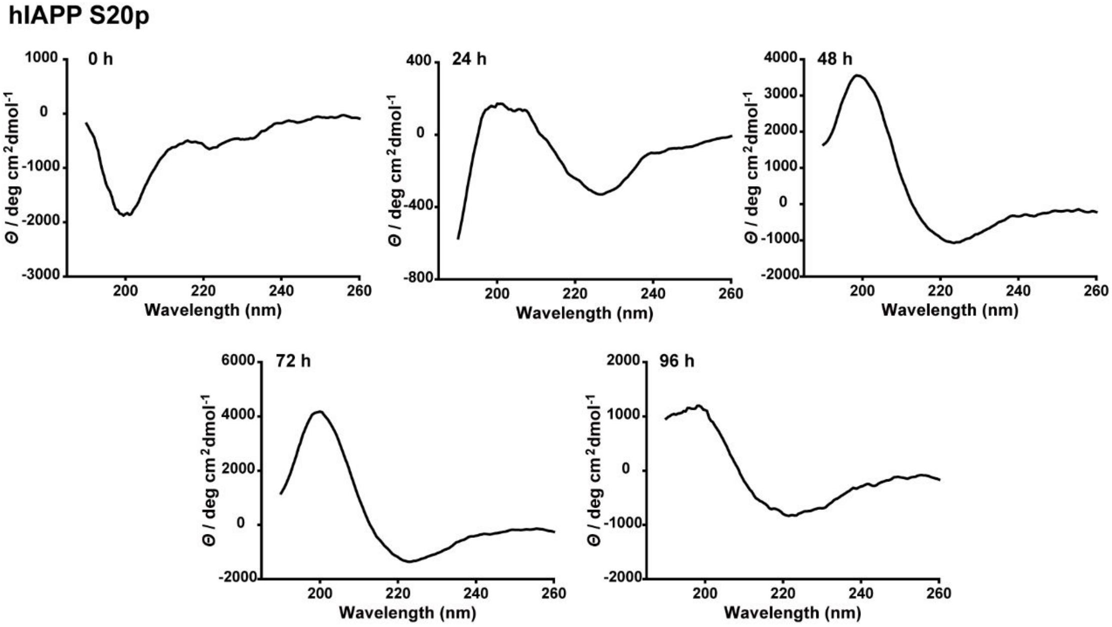

Figure S10. CD spectra of hIAPP S20p at various equilibration times.

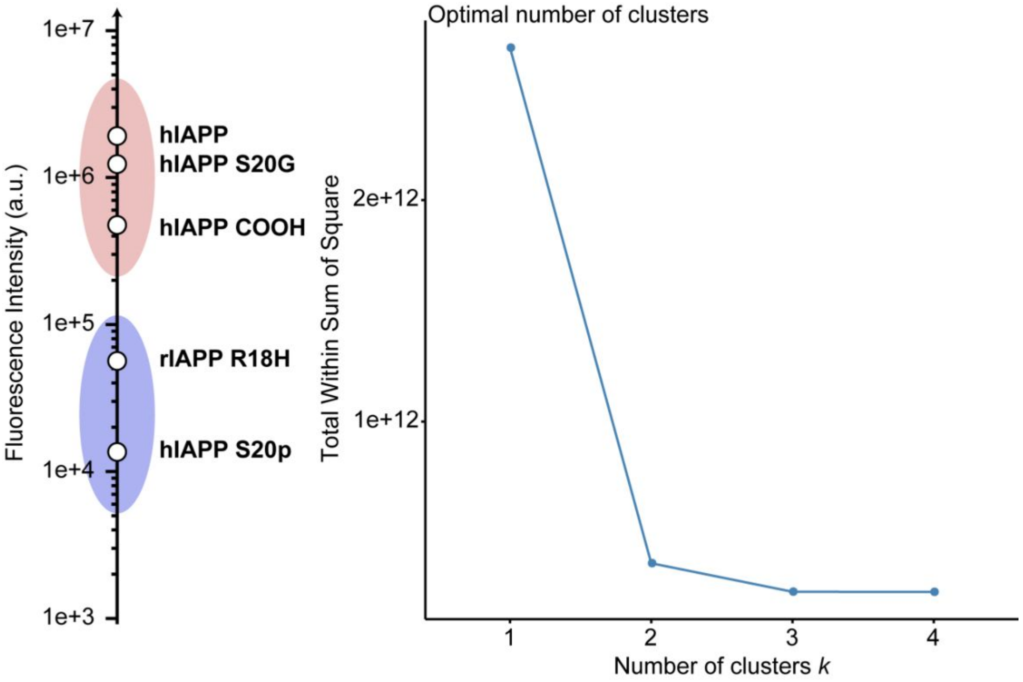

Figure S11. *K*-means clustering of the intensity of ThT fluorescence emitted from the solutions of hIAPP and its variants at 72 hours. The optimal number of clusters can be identified at the 'elbow' point, where the reduction in the total within sum of square begins to level off, which is at k =2.

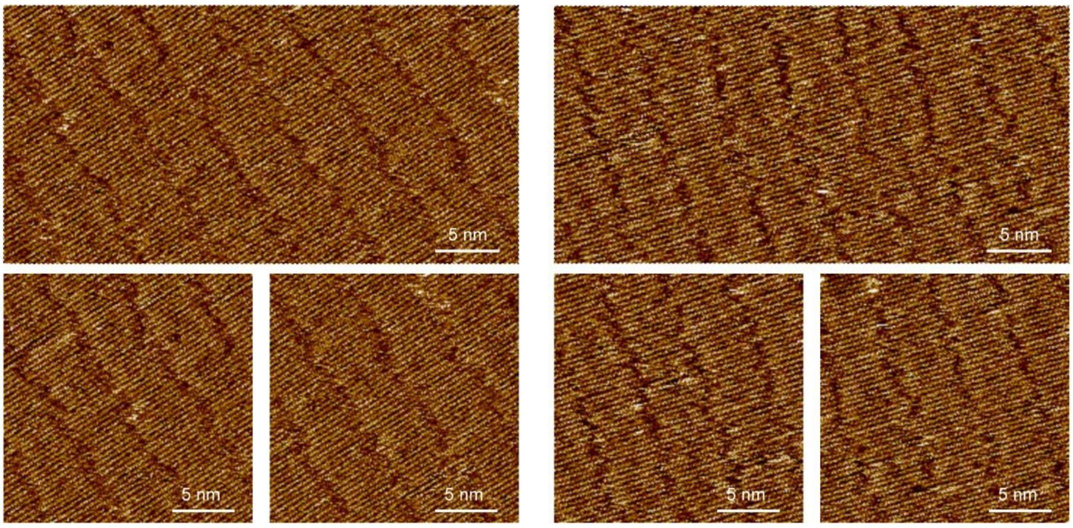

Figure S12. STM images of hIAPP assemblies on HOPG surfaces to show the reproducibility of the measurements. Tunneling conditions: bias voltage of 459.9 mV and tunneling current of 198.1 pA.

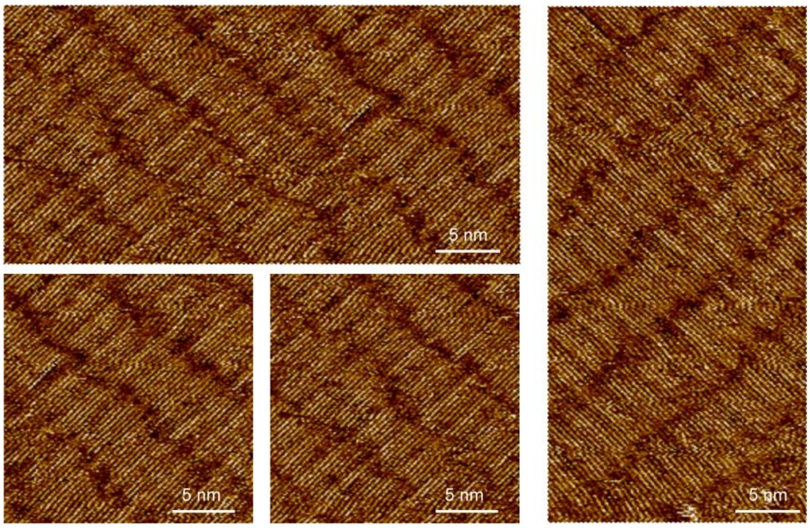

Figure S13. STM images of hIAPP S20G assemblies on HOPG surfaces to show the reproducibility of the measurements. Tunneling conditions: bias voltage of 499.9 mV and tunneling current of 198.1 pA.

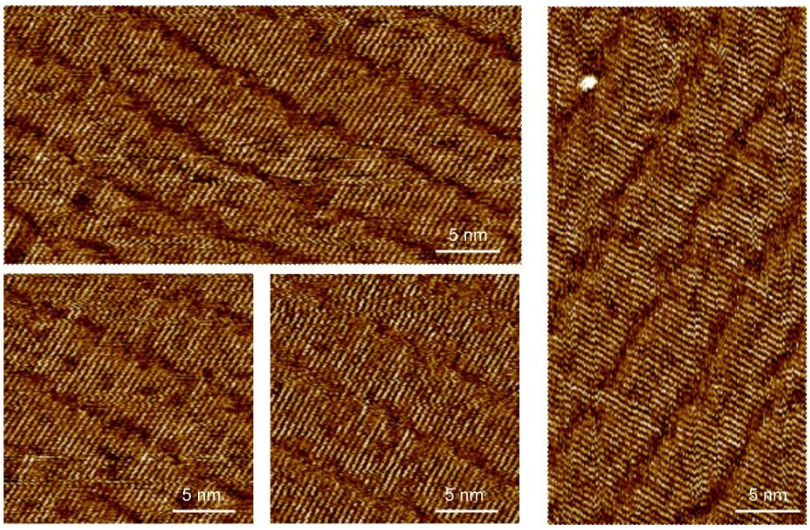

Figure S14. STM images of hIAPP COOH assemblies on HOPG surfaces to show the reproducibility of the measurements. Tunneling conditions: bias voltage of 500.0 mV and tunneling current of 198.1 pA.

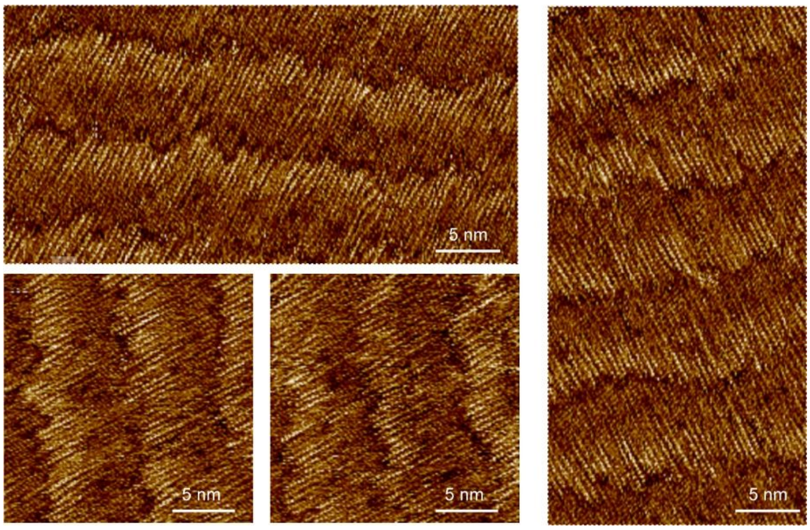

Figure S15. STM images of hIAPP S20p assemblies on HOPG surfaces to show the reproducibility of the measurements. Tunneling conditions: bias voltage of 549.9 mV and tunneling current of 198.1 pA.

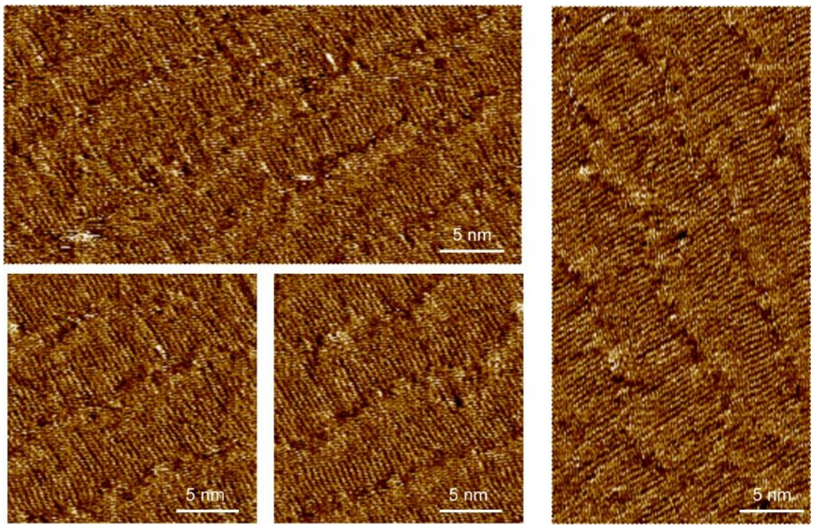

Figure S16. STM images of rIAPP R18H assemblies on HOPG surfaces to show the reproducibility of the measurements. Tunneling conditions: bias voltage of 499.8 mV and tunneling current of 198.1 pA.

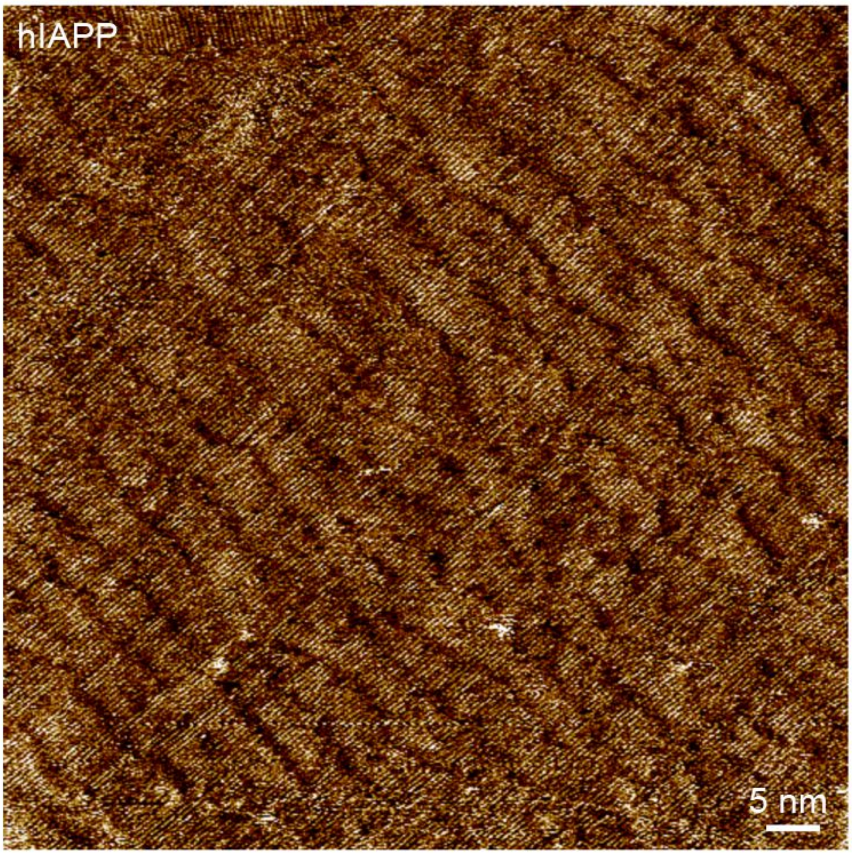

Figure S17. STM images (80 nm×80 nm) of hIAPP assemblies on HOPG surfaces. Tunneling conditions: bias voltage of 499.9 mV and tunneling current of 198.1 pA.

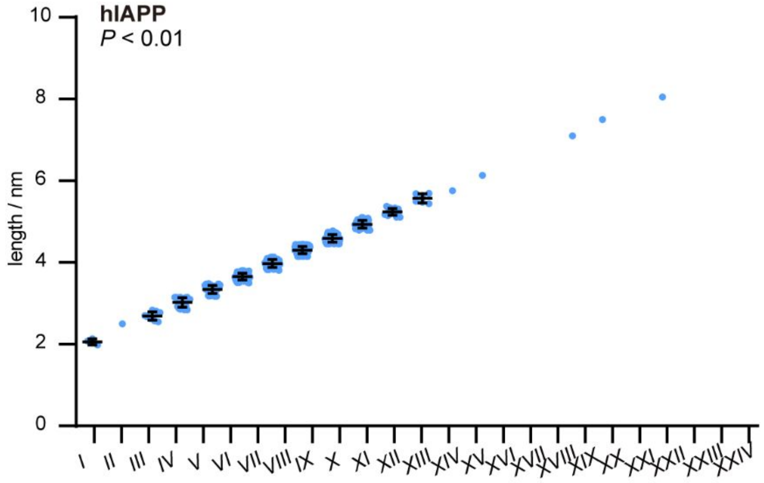

Figure S18. Length distribution of the hIAPP β-strands determined from the STM images.

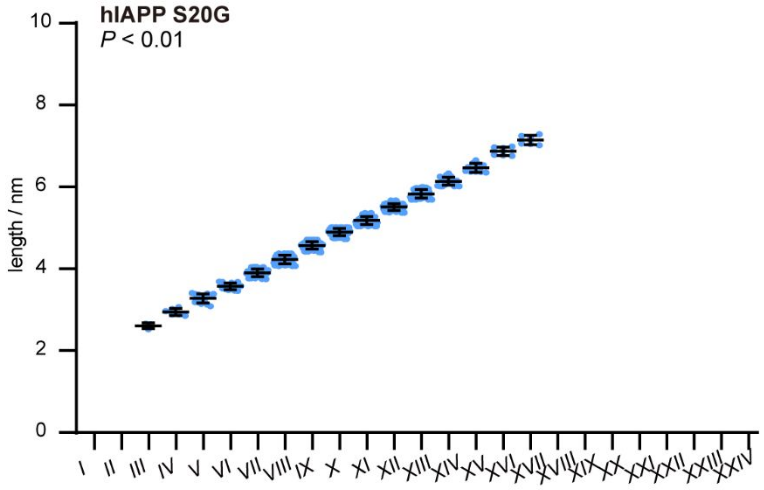

Figure S19. Length distribution of the hIAPP S20G β-strands determined from the STM images.

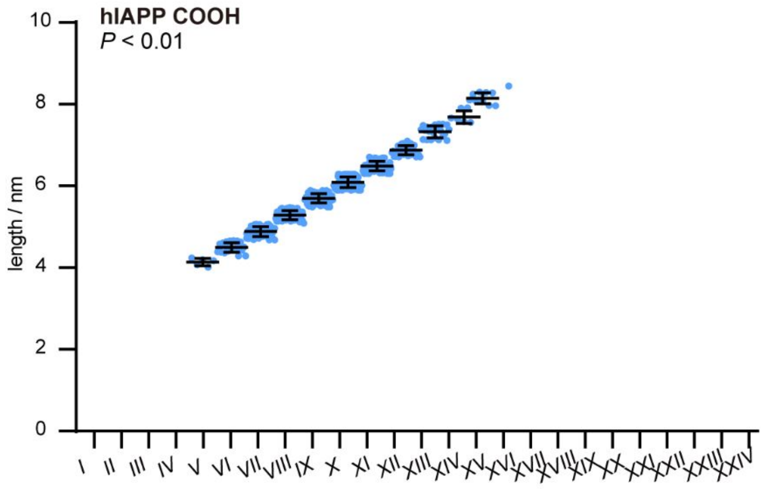

Figure S20. Length distribution of the hIAPP COOH β-strands determined from the STM images.

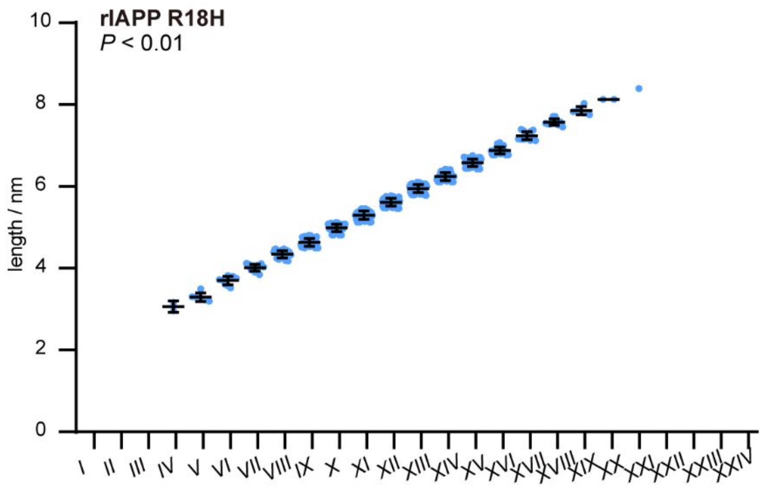

Figure S21. Length distribution of the rIAPP R18H β-strands determined from the STM images.

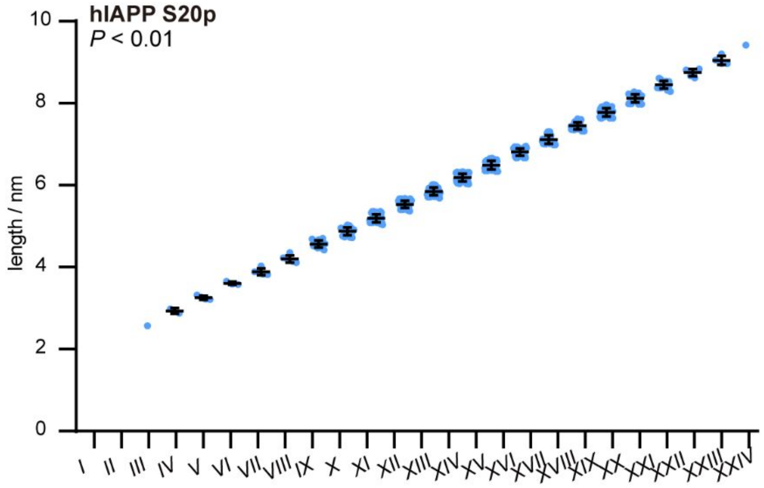

Figure S22. Length distribution of the hIAPP S20p β-strands determined from the STM images.

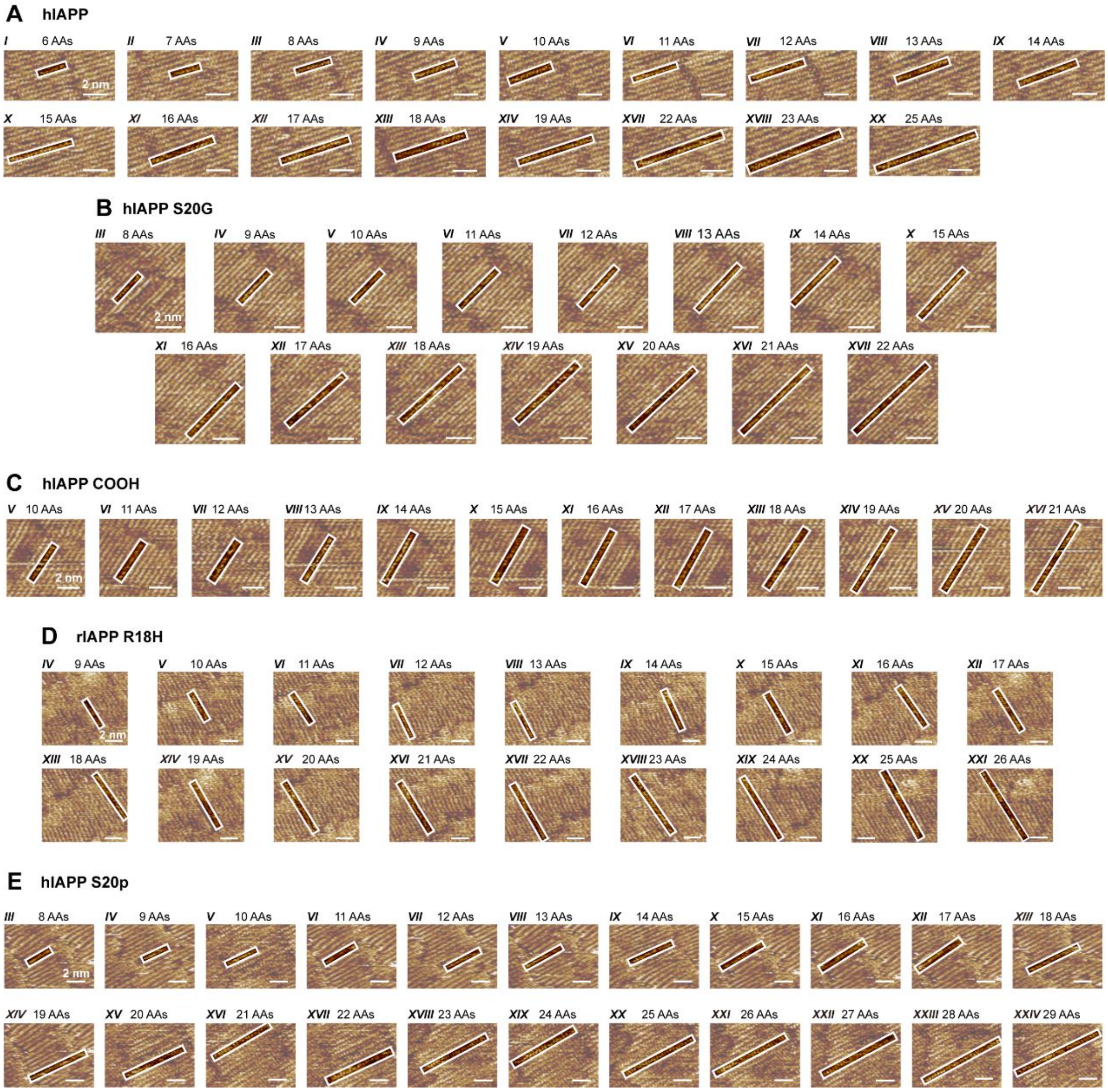

Figure S23. Representative STM images for each conformational sub-state. Scale bars represent 2 nm. (A) hIAPP, (B) hIAPP S20G, (C) hIAPP COOH, (D) rIAPP R18H, and (E) hIAPP S20p.
